# Adaptive Humor Styles as Predictors of Post-Traumatic Growth Factors

**DOI:** 10.3390/bs16071131

**Published:** 2026-07-06

**Authors:** Gert Kruger

**Affiliations:** Department of Psychology, University of Johannesburg, Auckland Park, Johannesburg 2006, South Africa; gkruger@uj.ac.za

**Keywords:** humor styles, post-traumatic growth, self-enhancing humor, structural equation modelling, cognitive reappraisal, trauma

## Abstract

This study examined whether adaptive humor styles, affiliative and self-enhancing, predict the five dimensions of post-traumatic growth (PTG) in a sample of South African undergraduate students who had experienced trauma. A cross-sectional SEM design was used with a criterion sample of 194 South African undergraduate students (72.7% female; *M* = 21.06 years, *SD* = 1.84) who had experienced a traumatic event between one and five years before participation. Self-enhancing humor was a significant positive predictor of all five PTG dimensions (Relating to Others, New Possibilities, Personal Strength, Spiritual Change, and Appreciation of Life), with standardized path coefficients ranging from β = 0.324 to β = 0.477. The measurement model demonstrated acceptable fit, though CFI (0.870) fell marginally below the conventional 0.90 threshold, likely due to model complexity. Affiliative humor did not independently predict any PTG dimension, with its zero-order correlations accounted for by shared variance with self-enhancing humor. These findings suggest that the emotion regulation and cognitive reappraisal functions of self-enhancing humor may facilitate growth across multiple domains following trauma, and have implications for therapeutic interventions aimed at promoting PTG.

## 1. Introduction

Exposure to trauma is a near-universal human experience, but individuals’ responses to trauma exposure can differ markedly. In South Africa the legacy of apartheid and ongoing structural inequality have resulted in exceptionally high rates of exposure to trauma, particularly in Black African communities (Atwoli et al., 2013). For example, in a sample of Black South African students, 97.6% of respondents indicated experiencing a traumatic event before entering university, with physical assault (69.3%), vehicular accidents (64.7%), and sudden death of a significant other (61.8%) being the top three reported experiences (Padmanabhanunni, 2020). Results from a longitudinal study following 2000 children from birth indicated that, by the age of 22, 99% of the children had experienced or witnessed violence in their homes, schools or communities (Richter et al., 2018).

Trauma is broadly understood as exposure to events that are overwhelming and uncontrollable. Tedeschi et al. (2018) emphasized that the definition of trauma should also include the fact that this event fundamentally challenges an individual’s sense of safety, meaning, and personal worth. While some experience prolonged distress, others report positive personal changes, known as post-traumatic growth (PTG), after experiencing highly challenging life events (Tedeschi & Calhoun, 1996, 2004).

### 1.1. Post-Traumatic Growth

Tedeschi and Calhoun (1996) proposed that PTG encompasses change across five distinct domains. *Relating to Others* refers to greater compassion, closeness, and willingness to express emotions. This domain involves the disruption and subsequent rebuilding of relational assumptions. *New Possibilities* involves the development of new interests and openness to change, which arises from the reorganization of goals and identity after schema disruption. *Personal Strength* involves increased self-reliance and an appreciation of one’s own resilience, which emerges from recognizing that one has survived what was previously believed to be unsurvivable. *Spiritual Change* refers to deepened spirituality or existential understanding, including a reassessment of meaning, purpose, and worldview. Finally, *Appreciation of Life* reflects a changed evaluation of everyday experiences and a greater gratitude for life. Because these domains cross interpersonal, intrapersonal, and philosophical dimensions, it is possible that different psychological processes are associated with growth in each.

Tedeschi and Calhoun (2004) postulated that PTG emerges after a traumatic event disrupted an individual’s assumptive world, including beliefs about safety, meaning, and personal worth. This disruption leads to the experience of rumination as the individual struggles to process the discrepancy between the event and existing assumptions or schemas. When this rumination gives way to more deliberate attempts to find meaning in the experience and to reconstruct a coherent worldview, PTG is experienced. It is this deliberate cognitive engagement, rather than the traumatic experience itself, that is associated with PTG across the five proposed domains. Studies have identified several facilitators, including resilience, optimism, social support, and emotion regulation (Henson et al., 2021; Prati & Pietrantoni, 2009). A psychological resource that appears to have received comparatively little attention as a predictor of PTG is humor.

### 1.2. Humor Styles

Humor has been associated with various positive psychological and physiological outcomes, including resilience, stress reappraisal, social facilitation, subjective well-being, and constructive coping (Amjad & Dasti, 2022; Martin & Ford, 2018; Richards & Kruger, 2017; Samson & Gross, 2012). All humor use is not, however, beneficial. Martin et al. (2003) proposed that healthy psychological outcomes are associated with both the use of positive humor and the absence of negative humor. In this regard, Martin et al. (2003) identified four styles of humor use, two of which are associated with beneficial outcomes (affiliative and self-enhancing humor) and two with maladaptive outcomes (aggressive and self-defeating humor).

*Affiliative humor* is used to promote interpersonal relationships, reduce interpersonal tension, and amuse others in ways that are warm and non-threatening. Through its role in reducing interpersonal tension and increasing closeness, affiliative humor actively maintains and deepens existing relationships and creates conditions for greater emotional expressiveness and compassion toward others (Martin et al., 2003; Rnic et al., 2016).

*Self-enhancing humor* refers to a humorous perspective on life, even under conditions of stress or adversity. This humor style has been found to play a role in the regulation of emotion which enables individuals to find amusement in difficult circumstances without minimizing the significance of such events (Ford et al., 2017; Kuiper & Leite, 2010; Martin et al., 2003). Beyond its stress-buffering function, self-enhancing humor reflects a broader existential orientation toward life, an ability to maintain perspective and find meaning even in the face of suffering, that extends beyond situational coping into a general reappraisal of one’s circumstances and priorities (Fu et al., 2024; Kuiper & Leite, 2010; Rnic et al., 2016).

Experimentally, self-enhancing humor has also been shown to provide better cognitive reappraisal than conventional reappraisal in terms of increasing positive affect and reducing negative affect (Fu et al., 2024; Samson & Gross, 2012; Samson et al., 2014). This seems to indicate that the incongruity-resolution mechanism associated with self-enhancing humor provides stronger emotional distancing than deliberate reappraisal alone (Fu et al., 2024). Similarly, evidence from neuropsychological studies indicated that humorous reappraisal produced deeper cognitive reconstruction and more durable emotion regulation than conventional reappraisal (Wu et al., 2021). Both affiliative and self-enhancing humor have been associated with positive psychological outcomes, including higher self-esteem, increased wellbeing, optimism, quality of life, and more effective coping (Čekrlija et al., 2025; Stieger et al., 2011; Zhao et al., 2014).

The remaining two styles, aggressive and self-defeating humor, are associated with maladaptive psychological outcomes (Jiang et al., 2020; Schneider et al., 2018). *Aggressive humor* is used to embarrass, degrade, threaten or manipulate others for amusement and to enhance social position. *Self-defeating humor* involves the use of humor to degrade or embarrass oneself in order to gain social approval (Martin et al., 2003). Meta-analytic studies reported that both these styles have been associated with increased psychological distress, reduced wellbeing, and maladaptive coping (Jiang et al., 2020; Schneider et al., 2018). These humor styles were excluded from the current study^1^ as they have been found to be positively related to avoidance coping, cognitive distortions, decreased cognitive reappraisal, and maladaptive rumination (Perchtold et al., 2019; Poncy, 2017; Rnic et al., 2016; Schneider et al., 2018) and have not been theoretically linked to the reappraisal and meaning-making processes central to PTG.

### 1.3. PTG and Adaptive Humor

Little is known about the relationship between adaptive humor styles and post-traumatic growth. Boerner et al. (2017) reported no significant associations between any of the four humor styles and PTGI total scores across two UK samples. They did, however, find that self-enhancing humor was positively associated with positive post-trauma change when the latter was measured on the Changes in Outlook Questionnaire (CiOQ-P). The authors suggested that these findings could indicate that the relationship between self-enhancing humor and post-trauma adjustment is dependent on how growth is operationalized.

In a recent study, Goncharov (2025) reported a significant positive association between self-enhancing humor and the total PTGI score in a Central European sample. The remaining three humor styles did not yield any significant associations. In exploratory post hoc analyses focusing on the PTGI sub-scales, Goncharov (2025) found that self-enhancing humor predicted New Possibilities and Appreciation of Life, with the author noting that the former may benefit from self-enhancing humor’s cognitive reappraisal role and the latter from its perspective-shifting function.

Theoretically, adaptive humor styles and PTG share psychological mechanisms, as well as the cognitive processes Tedeschi and Calhoun (2004) identified as driving growth. For example, self-enhancing humor involves finding alternative, less threatening interpretations of difficult situations (Martin et al., 2003). This process relates to the cognitive reappraisal thought to facilitate meaning-making after the experience of trauma (Perchtold et al., 2019). Self-enhancing humor has also been associated with the maintenance of positive affect while experiencing stress (Braniecka et al., 2019; Ford et al., 2016). Fredrickson (2001) proposed that such a skill could broaden cognitive and behavioral resources in ways that may support reconstruction and meaning-making. Additionally, self-enhancing humor has been linked to reduced anxiety and better adjustment after experiencing stressful events (Vaughan et al., 2014), suggesting that this humor style could also be relevant to the broader adaptive processes associated with PTG.

In contrast, affiliative humor may facilitate PTG through an interpersonal pathway separate from cognitive reappraisal. By managing social bonds and reducing interpersonal tension (Martin et al., 2003), this humor style may enhance perceived social support, which, in itself, has been shown to be a predictor of PTG (Prati & Pietrantoni, 2009; Tedeschi & Calhoun, 2004). Based on these functions, it was expected that the predictive role of affiliative humor would be limited to interpersonal aspects of PTG, particularly Relating to Others.

### 1.4. Current Study

The present study builds on the results reported by Boerner et al. (2017) and Goncharov (2025) by examining whether the two adaptive humor styles predict each of the five PTGI subscales separately. The five domains proposed by Tedeschi and Calhoun (2004) encompass both interpersonal change (Relating to Others), as well as intrapersonal adjustment (New Possibilities, Personal Strength) and existential growth (Spiritual Change, Appreciation of Life). Interpersonal change may be facilitated by the social functions of affiliative humor, whereas intrapersonal change and existential growth may be linked to self-enhancing humor’s reappraisal functions. These two styles of humor may therefore differ in their associations with the five domains. The present study examined the possible relationships between PTG and the two adaptive humor styles by focusing on a criterion sample of South African undergraduate students who had experienced a traumatic event between one and five years prior to participation.

Two hypotheses were tested:

**H1.**  *Given its role in cognitive reappraisal, emotion regulation, and existential meaning-making, self-enhancing humor was hypothesized to positively predict all five PTG dimensions.*

**H2.**  *Given that affiliative humor functions primarily through social facilitation and relationship maintenance, rather than through intrapersonal reappraisal, it was hypothesized that this humor style would positively predict the Relating to Others dimension only.*

## 2. Materials and Methods

### 2.1. Participants and Procedure

A total of 234 undergraduate students completed the survey. 40 students indicated absence of trauma and were excluded from analyses, resulting in a purposive criterion sample of 194 participants who complied with the remaining inclusion criterion which required that they had experienced a traumatic event between one and five years prior to the study. Participants were Indian (4.6%), Colored (6.6%), Black (80.2%) and White (8.6%)^2^. The ethnic composition of the sample closely reflected South Africa’s national demographic profile, in which Black Africans constitute approximately 81% of the population (Statistics South Africa, 2024). Participants were aged between 18 and 33 years (*M* = 21.06, *SD* = 1.84) and consisted of 141 (72.7%) females and 53 (27.3%) males. Although 93.8% of the participants reported a first language other than English, the survey was still administered in English, as enrolment into the institution required a minimum level of English proficiency, whether as a first or additional language, in students’ final school year. Additionally, English is the institution’s established medium of instruction.

In South Africa children are required to learn two languages as part of their schooling. Most parents prefer English, as it is viewed as the language of upward mobility and access to higher education. English is typically introduced as a First Additional Language from Grade 1 (ages 6 to 7), becomes the medium of instruction from Grade 4 (ages 9 to 10), and was the dominant language of learning and teaching at 67.7% of schools nationally in 2011 (Wildsmith-Cromarty & Balfour, 2019). By the time students enter university, most will therefore have received over a decade of structured English language education. Data were collected in August 2017.

Students were advised about the study and invited to participate. No incentive was offered, and students who chose not to participate did not face any academic or other consequences. Participants were requested to complete the questionnaires on the university’s online student portal. Although login was required, completion was entirely anonymous and responses could not be linked to a particular participant. Before completing the questionnaires, participants were required to indicate informed consent.

### 2.2. Measures

In order to gather demographic information, a biographical questionnaire was used in which participants were required to indicate their age, gender, ethnic affiliation, and first language. Participants were also asked to indicate whether they had experienced a traumatic event (“Have you ever experienced an event that you considered to be traumatic?”), to specify when the event occurred (“If yes, when did this event occur (year and month)?”, and to provide a brief description of the nature of the event (“Please briefly describe this event:”). Events were not screened against a formal diagnostic criterion, consistent with research demonstrating that PTG can follow a broad range of stressful life events beyond those meeting clinical trauma thresholds (Özgüç et al., 2022). The traumatic events were broadly categorized based on participants’ brief descriptions. Table 1 displays the frequencies and types of trauma the participants disclosed.

Inclusion in the study required that the traumatic event had occurred between one and five years before the study. The mean time elapsed between the reported event and participation in this study was 1.92 years (*SD* = 1.37). The lower limit of one year was intended to allow sufficient time for the core PTG process, deliberate cognitive processing, to emerge (Tedeschi & Calhoun, 2004). Gendre et al. (2025) pointed out that, although positive changes had been reported as early as two weeks post-trauma (Frazier et al., 2001; Kleim & Ehlers, 2009), it is possible that these reports are susceptible to cognitive biases, such as positive attention bias and memory bias. Participants’ reports may, therefore, reflect illusory, rather than constructive growth (Zoellner & Maercker, 2006).

An upper limit of five years was chosen with the intention to reduce the possibility that life changes were unrelated to the reported trauma. It was also possible that the reported changes could be substantially affected by retrospective memory distortion, given that PTG trajectories tend to stabilize within approximately two years after the traumatic event (Gendre et al., 2025).

Two self-report measures were used to assess humor styles and post-traumatic growth.

#### 2.2.1. The Humor Styles Questionnaire (HSQ; Martin et al., 2003)

The HSQ consists of four scales of eight items each, two of which are other-directed (affiliative and aggressive humor) and two which are self-directed (self-enhancing and self-defeating humor). Responses are measured on a scale ranging from 1 (totally disagree) to 7 (totally agree). Affiliative humor is assessed by questions such as, “I enjoy making people laugh”, whereas questions such as, “It is my experience that thinking about some amusing aspect of a situation is often a very effective way of coping with problems”, assess self-enhancing humor.

The HSQ has shown satisfactory internal consistency on all scales, with Cronbach’s alphas of 0.80 for affiliative humor and 0.81 for self-enhancing humor reported (Martin et al., 2003). In the current study the Cronbach’s alphas were 0.81 and 0.83 for the affiliative and self-enhancing humor, respectively (See Table 2).

#### 2.2.2. The Post-Traumatic Growth Inventory (PTGI; Tedeschi & Calhoun, 1996)

The Post-Traumatic Growth Inventory (PTGI; Tedeschi & Calhoun, 1996) is used to measure the degree to which trauma survivors experience positive changes due to their efforts to cope with a trauma and its consequences (Calhoun et al., 2000). The scale consists of 21 items which are divided into five subscales, namely Relating to Others (7 items), e.g., “I have a greater sense of closeness with others”; New Possibilities (5 items), e.g., “I developed new interests”; Personal Strength (4 items), e.g., “I have a greater feeling of self-reliance”; Spiritual Change (2 items), e.g., “I have a stronger religious faith”; and Appreciation of Life (3 items), e.g., “I changed my priorities about what is important in life”. Participants are requested to indicate the extent to which each positive change occurred in their lives as a consequence of their traumatic experience. Responses are measured on a 6-point Likert-type scale, ranging from 0 (I did not experience this change as a result of my crisis) to 5 (I experienced this change to a very great degree as a result of my crisis).

A very good Cronbach’s alpha of 0.90 has been reported for the PTGI (Taku et al., 2008). In South Africa, where the current study was conducted, the PTGI has previously demonstrated a Cronbach’s alpha of 0.95 for the total scale (Walker-Williams et al., 2012). Table 2 indicates that the Cronbach’s alphas and McDonald’s omegas ranged between 0.63 and 0.82 for the current study. The internal consistency estimates of the Spiritual Change (α = 0.681) and Appreciation of Life (α = 0.633) subscales were marginally below the conventional 0.7 threshold. This is most likely due to the brevity of these subscales (Cortina, 1993), with the former consisting of two and the latter of three items. Notably, Tedeschi et al. (2017) recognized that the two items of the Spiritual Change subscale may not be sufficient nor adequately assess growth in cultures that are more secular. An expanded version of the PTGI (the PTGI-X) was subsequently developed, in which the Spiritual Change subscale was renamed to Spiritual–Existential Change (SEC), containing six items, which now include a focus on existential growth. The Appreciation of Life subscale was not expanded in the PTGI-X.

The lower alphas found in the current study for these scales are consistent with similar alphas (0.67–0.75) reported in other studies (Cheonga & Pieters, 2022; Taku et al., 2008; Tedeschi & Calhoun, 1996), thus indicating that the low values were not specific to the sample in this study. This is further supported by the fact that 98.5% of participants reported adherence to some form of spirituality, particularly Christianity, thereby negating the possibility of secularity influencing internal consistency. The McDonald’s omega values were also almost identical to the Cronbach’s alphas for both subscales (Spiritual Change: ω = 0.68; Appreciation of Life: ω = 0.65). This indicates that a single common factor dominated variance and suggests sufficient construct homogeneity, despite the low alpha estimates (Dunn et al., 2014; McNeish, 2018). Lastly, mean inter-item correlations were calculated for these two subscales and compared to the range (0.15 to 0.50) recommended by Clark and Watson (2019). Values of 0.52 and 0.37 were found for the Spiritual Change and Appreciation of Life subscales, respectively. Thus, the latter falls well within this range, and the former marginally exceeds it.

### 2.3. Data Analysis

Analyses were conducted in R 4.5.1 (R Core Team, 2025) using the lavaan package (Rosseel, 2012) for structural equation modelling (SEM) and the psych package (Revelle, 2024) for reliability estimation. Multivariate normality was assessed with the MVN package (Korkmaz et al., 2014) using Mardia’s (1970) skewness and kurtosis tests.

A CFA measurement model was first estimated to evaluate the latent factor structure of the seven constructs and to establish adequate measurement quality before evaluating structural relationships between the humor style predictors and PTG dimensions. A full structural model was then specified where each of the five PTG latent factors was regressed on both affiliative and self-enhancing humor (i.e., the fully saturated structural model). Residual covariances among the five PTG factors were freed as the dimensions represent theoretically related facets of a common growth process measured with a single instrument in a single session (Kline, 2016).

The association between the two humor styles and post-traumatic growth at the construct level was also explored in a separate analysis. A SEM analysis, where a single error-corrected total PTGI composite (the mean of all 21 items) served as the outcome indicator, was performed. The indicator’s residual variance was set to Var(*PTG_Total_*) × (1 − α) = 0.422 × (1 − 0.888) = 0.047 to account for measurement error (Bollen, 1989). The full latent factor measurement model from the primary analysis was retained.

Aggressive and self-defeating humor were excluded from the primary analyses, based on theoretical grounds and previous research findings. To provide a more complete picture, an exploratory supplementary SEM was conducted in which all four humor styles were entered simultaneously as latent predictors of each PTG dimension and total PTGI. Results are reported in Supplementary Materials.

Due to the presence of multivariate non-normality in the PTGI and HSQ subscale scores (see Section 3.3 below), the Maximum Likelihood with Robust Standard Errors (MLR) estimator was utilized for all analyses. This approach produced Satorra–Bentler-scaled χ^2^ statistics, as well as robust estimates for CFI, TLI, and RMSEA. Model fit was evaluated against the following conventional thresholds: CFI ≥ 0.90 (adequate)/≥ 0.95 (excellent); TLI ≥ 0.90; RMSEA ≤ 0.08 (acceptable)/≤ 0.06 (close fit); SRMR ≤ 0.08 (Hu & Bentler, 1999; Kline, 2016).

### 2.4. Power Analysis

An *a priori* power analysis for the obtained sample (*N* = 194) was performed. At the path level, power was estimated for a small-to-medium structural path coefficient of β = 0.30 (*f*^2^ = 0.10; Cohen, 1988) using the semPower package (Moshagen & Erdfelder, 2016). Results indicated that a sample of *N* = 81 was required to achieve 80% power (α = 0.05, two-tailed).

At the model level, a sample of *N* = 45 was required to achieve 80% power to distinguish close fit (RMSEA = 0.05) from poor fit (RMSEA = 0.08), given the degrees of freedom (*df* = 608) (MacCallum et al., 1996). The sample in this study provided power of 1.000 for this test. The study therefore had adequate power for both path-level and model-level inferences at conventional effect sizes.

## 3. Results

### 3.1. Trauma Types and Frequencies

The range of traumatic experiences reported by participants appears in Table 1. The most commonly reported events were death of a loved one (*n* = 52, 26.8%) and being robbed (*n* = 52, 26.8%), followed by experiencing a serious accident or health problem (*n* = 37, 19.1%).

### 3.2. Preliminary Analyses

Subscale means, standard deviations, Cronbach’s alpha (α), McDonald’s omega (ω) values, and bivariate correlations are shown in Table 2. Internal consistency estimates ranged from acceptable to good across all subscales. PTG-IV (Spiritual Change; α = 0.681) and PTG-V (Appreciation of Life; α = 0.633) had the lowest estimates, attributable in part to their short length (two and three items, respectively). However, their inclusion as latent factors in SEM partially compensates for the reliability attenuation associated with short scales. Both HSQ subscales demonstrated good reliability.

Notably, self-enhancing humor showed statistically significant positive correlations with all five PTG subscales (*r* = 0.214–0.284, all *p* < 0.01). By contrast, affiliative humor only showed a significant correlation with PTG-I: Relating to Others (*r* = 0.189, *p* = 0.008). The two humor subscales correlated moderately positively with each other (*r* = 0.525, *p* < 0.001). PTG subscale intercorrelations were moderate to large (*r* = 0.281–0.605), reflecting the multidimensional yet related nature of post-traumatic growth.

For exploratory findings regarding aggressive and self-defeating humor, see Supplementary Materials.

### 3.3. Multivariate Normality

Mardia’s (1970) test indicated significant multivariate skewness for both the PTG subscale scores (χ^2^ = 103.57, *p* < 0.001) and the HSQ subscales (χ^2^ = 11.84, *p* = 0.019). While multivariate kurtosis was non-significant for the PTG subscales (*z* = 1.75, *p* = 0.080), it was found to be significant for the HSQ subscales (*z* = 2.06, *p* = 0.039). Both skewness statistics were statistically significant; however, the magnitude of violation was small. The MLR estimator with robust errors was therefore used, as it is considered sufficient to correct for parameter bias at this level of non-normality (Satorra & Bentler, 1994).

### 3.4. Measurement Model (Confirmatory Factor Analysis)

A seven-factor CFA was estimated, where affiliative and self-enhancing humor, as well as the five PTG latent factors (I–V), were specified. All 37 indicators loaded onto their designated factors. No cross-loadings were specified. Residual covariances among all five PTG factors were freed to account for shared method variance. The MLR estimator was used throughout.

Model fit was acceptable: χ^2^(608) = 893.40, *p* < 0.001; CFI = 0.870; TLI = 0.858; RMSEA = 0.051, 90% CI [0.043, 0.057]; SRMR = 0.074. Fit indices fell slightly below the CFI ≥ 0.90 threshold, though RMSEA and SRMR remained within acceptable ranges given the model’s complexity (37 indicators, seven latent factors) (Kenny & McCoach, 2003; Shi et al., 2019). All standardized factor loadings were statistically significant (*p* < 0.001 or *p* = 0.005), ranging from 0.280 to 0.837 (see Table 3).

### 3.5. Structural Model

#### 3.5.1. Model Specification

The structural model regressed each of the five PTG latent factors on both affiliative and self-enhancing humor simultaneously (saturated structural model with all ten paths freely estimated). The measurement structure (indicators and loadings) was identical to the CFA. Free residual covariances among the five PTG factors were retained. The latent correlation between affiliative and self-enhancing humor was freely estimated. All parameters were estimated using MLR.

#### 3.5.2. Model Fit

Fit indices for both the measurement model and the structural model are reported in Table 4. As the structural model is saturated (all predictor-to-outcome paths are freely estimated), its fit is mathematically equivalent to that of the CFA measurement model.

#### 3.5.3. Structural Path Coefficients

Standardized and unstandardized path coefficients for all ten structural paths are presented in Table 5. The latent correlation between affiliative and self-enhancing humor was *r* = 0.593 (*p* < 0.001).

Self-enhancing humor was a significant positive predictor of all five PTG dimensions: Relating to Others (β = 0.324, *p* = 0.008), New Possibilities (β = 0.460, *p* < 0.001), Personal Strength (β = 0.453, *p* < 0.004), Spiritual Change (β = 0.477, *p* < 0.001), and Appreciation of Life (β = 0.430, *p* = 0.002). Standardized coefficients ranged from 0.324 to 0.477, indicating small to moderate positive effects. Sensitivity analyses confirmed that the study had 80% power to detect standardized path coefficients of β ≈ 0.32–0.44 at *N* = 194. All five self-enhancing humor paths exceeded this threshold (β = 0.324–0.477), confirming that significant findings were not products of an oversensitive model.

Affiliative humor did not significantly predict any PTG dimension (all *p* > 0.25). Standardized coefficients for affiliative humor were also small and non-significant across all five PTG outcomes (β = 0.009 to −0.142; all *p* > 0.25). The affiliative humor coefficients were consistently negative (except for PTG-I, β = 0.009) when controlling for self-enhancing humor, despite the positive zero-order correlation between affiliative humor and PTG-I (*r* = 0.189). This outcome possibly reflects suppression due to the high latent intercorrelation between the two predictors (*r* = 0.593). Variance inflation factors for affiliative humor (VIF = 1.54) and self-enhancing humor (VIF = 1.54) indicated that multicollinearity, while present, was not at a level that would affect parameter estimation.

Post hoc power for affiliative humor was low (0.05–0.20), which might suggest insufficient power to detect existing effects. However, post hoc power is a direct function of the observed effect and is therefore uninformative beyond the *p*-value itself (Hoenig & Heisey, 2001).

**Figure 1:**
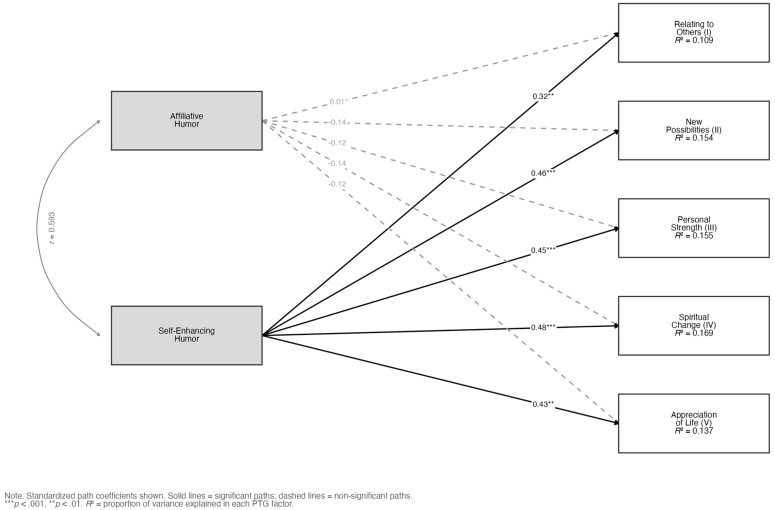
Structural Model: Adaptive Humor Styles Predicting Post-traumatic Growth.

The structural model explained 10.9% of the variance in PTG-I (Relating to Others), 15.4% in PTG-II (New Possibilities), 15.5% in PTG-III (Personal Strength), 16.9% in PTG-IV (Spiritual Change), and 13.7% in PTG-V (Appreciation of Life). Structural paths between affiliative humor, self-enhancing humor, and the five PTG latent factors, including standardized coefficients, significance markers, *R*^2^ values, and the latent correlation between the two humor predictors, are illustrated in Figure 1.

### 3.6. Total PTGI as Outcome

The additional SEM analysis confirmed that self-enhancing humor significantly predicted total PTGI (β = 0.455, *p* < 0.001). Affiliative humor did not yield significant results (β = −0.064, *p* = 0.526). The two predictors explained 17.7% of the variance in total PTG (see Table 6).

### 3.7. Exploratory Analysis: Extended Model Including Maladaptive Humor Styles

The exploratory model included all four humor styles (see Supplementary Materials for full results). Neither aggressive nor self-defeating humor was a significant predictor of any PTG domain, including the total score. Self-enhancing humor remained the sole significant predictor across all five PTG dimensions and the total PTGI in the exploratory model.

## 4. Discussion

This study examined whether affiliative and self-enhancing humor predict the five dimensions of post-traumatic growth proposed by Tedeschi and Calhoun (2004). Results supported the first hypothesis, which stated that self-enhancing humor would be a significant positive predictor of all five PTG dimensions. The second hypothesis was, however, not supported. Affiliative humor did not independently predict any dimension of PTG after controlling for self-enhancing humor.

### 4.1. Self-Enhancing Humor as a Predictor of PTG

Self-enhancing humor was a consistent and significant positive predictor of all five PTG dimensions, with standardized path coefficients ranging from β = 0.324 to β = 0.477. These results align with the notion that self-enhancing humor involves an emotion regulation strategy associated with cognitive reappraisal and reduced negative affect in the context of stressful events (Martin et al., 2003). Tedeschi and Calhoun (2004) proposed that deliberate cognitive processing and reappraisal of trauma-related beliefs are central mechanisms of growth. Self-enhancing humor may play a role in these types of reappraisal, thereby supporting trauma survivors in the reconstruction of their experiences towards growth.

Self-enhancing humor predicted growth across both interpersonally oriented domains, such as Relating to Others, and philosophically oriented domains, such as Spiritual Change and Appreciation of Life. This suggests that its role may be broad, rather than domain-specific. This breadth is consistent with the view that self-enhancing humor functions as a general cognitive and emotional resource rather than a situation-specific coping instrument (Fu et al., 2024). Fredrickson’s (2001) broaden-and-build theory supports this notion by indicating that positive emotions and the processes sustaining them, such as reappraisal through humor, can expand individuals’ cognitive and behavioral range. In particular, its reappraisal function aligns with Eissenstat et al.’s (2024) meta-analytic finding that positive emotion-focused coping was significantly associated with all PTG dimensions, whereas negative emotion-focused coping was not. Consistent with this mechanistic interpretation, Suriano and Valentino (2026) reported that positive humor facilitated tolerance for ambiguity, which in turn predicted greater resilience in a sample of Italian young adults. This sequential pathway aligns with the broad reappraisal and meaning-reconstruction processes central to PTG.

At the subscale level, Goncharov (2025) identified associations between self-enhancing humor and New Possibilities and Appreciation of Life. The author noted that New Possibilities might benefit from the cognitive reappraisal role of self-enhancing humor and Appreciation of Life from its perspective-shifting function. The findings in the present study are consistent with this interpretation but suggest that these two mechanisms might operate more broadly. Self-enhancing humor was found to be a significant predictor of all five PTG domains, including those characterized by a more interpersonal or existential nature. Thus, it seems that self-enhancing humor plays a general supportive cognitive and emotional resource role, rather than one linked to specific growth domains.

On a construct level, self-enhancing humor was a significant positive predictor of total PTG. This finding supports that of Goncharov (2025), who reported a similar result in a Central European sample. While this result does not align with the findings of Boerner et al. (2017), the inconsistency might be due to sample differences, such as cultural context or sample size, or due to methodological scope. Boerner et al. (2017) reported bivariate correlations, which were appropriate for their exploratory aims but cannot control for the predictive contributions of other humor styles when estimating each style’s unique association with PTG. The present study extended this approach by estimating the unique predictive contribution of each humor style while accounting for shared variance.

### 4.2. Affiliative Humor and Suppression

At the zero-order level, affiliative humor showed a small but significant positive correlation with Relating to Others (*r* = 0.189), suggesting a meaningful bivariate association.

However, once both predictors were included simultaneously in the structural model, the path coefficients for affiliative humor were near-zero or negative across all five PTG dimensions. While affiliative humor showed small positive zero-order correlations (*r* = 0.071 to 0.125) with New Possibilities, Personal Strength, Spiritual Change, and Appreciation of Life, its standardized path coefficients in the structural model were negative (β = −0.120 to −0.142), indicating classical suppression (MacKinnon et al., 2000).

Relating to Others showed a slightly different pattern. The path coefficient fell to near zero (β = 0.009), suggesting that the bivariate association between affiliative humor and Relating to Others was fully accounted for by its shared variance with self-enhancing humor (latent *r* = 0.593) rather than reversed by it. It seems, therefore, that affiliative humor’s zero-order associations with PTG reflected the variance it shared with self-enhancing humor, rather than any independent contribution.

The non-significant findings for affiliative humor are more likely to reflect truly negligible unique effects than being due to Type II error. Sensitivity analyses indicated a minimum detectable effect of β ≈ 0.32, and the contributions of affiliative humor fell well below this threshold.

On a construct level, affiliative humor was also not a significant predictor of total PTG. Affiliative humor functions mainly through social facilitation, tension reduction, and strengthening of interpersonal bonds (Henson et al., 2021; Martin et al., 2003) but does not relate directly to the intrapersonal cognitive reappraisal and meaning reconstruction that PTG theory identifies as central to growth (Tedeschi & Calhoun, 2004). Instead, affiliative humor may contribute to a social environment conducive to growth, but this contribution seems to function through its overlap with self-enhancing humor, rather than independently. Future research using larger samples could examine whether affiliative humor predicts PTG indirectly, for example, through social support or perceived closeness with others, rather than directly.

### 4.3. Negative Humor Styles

The exploratory analyses reported in Supplementary Materials confirmed that neither aggressive nor self-defeating humor significantly predicted any of the PTGI domains when all four humor styles were entered simultaneously. These results align with those reported by Goncharov (2025). It should be noted, however, that the aggressive humor scale yielded particularly poor internal consistency (α = 0.429), which led to unstable path coefficients. Also of note is that self-enhancing humor still emerged as the only significant predictor of all five PTGI domains.

### 4.4. Limitations and Future Directions

Some limitations of the present study should be noted. The sample consisted of undergraduate students, mainly female (72.7%), from a single South African university. These factors limit generalizability to other populations and cultural contexts. Future research with more diverse samples would be needed to assess whether the findings of this study replicate across ethnic groups.

The cross-sectional design further prevents causal inference. While the results are consistent with the interpretation that self-enhancing humor facilitates PTG, it is also possible that individuals who experience greater PTG develop a more self-enhancing humor style as a consequence of growth. Longitudinal designs would be necessary to establish directionality. PTG was assessed retrospectively, which could introduce recall bias, thereby limiting conclusions about the specific relationship between humor use and growth.

As an initial investigation into the relationship between adaptive humor styles and PTG dimensions, the present study followed a parsimonious design to establish a baseline understanding of these associations and to optimize statistical power for detecting the primary intra-psychic pathways of interest, before introducing additional complexity. Accordingly, several variables found to be associated with both humor use and PTG, such as resilience, optimism, and social support (Henson et al., 2021; Prati & Pietrantoni, 2009), were not included. Omitting such variables could inflate estimated path sizes, leading to an overestimation of the unique contribution of self-enhancing humor to PTG (Kline, 2016).

Similarly, the mechanisms through which self-enhancing humor may foster PTG were not examined in the current study. Mediators, such as cognitive reappraisal, benefit-finding, and deliberate rumination, are theoretically plausible (Perchtold et al., 2019; Tedeschi & Calhoun, 2004) and future studies could examine whether such variables mediate the relationship between self-enhancing humor and PTG. Moderators, such as trauma severity, time elapsed since the traumatic event, gender, and cultural context, may also influence the strength of the relationship between humor styles and PTG. It is recommended that future studies include covariates, mediators, and moderators to assist in establishing boundary conditions and mechanisms underlying the relationship between self-enhancing humor and PTG.

The study relied on self-report measures for both humor styles and PTG. As with all self-report instruments, the HSQ and PTG may be susceptible to biases, such as social desirability. HSQ items, in particular, may be vulnerable to self-enhancement, where participants could overestimate their use of adaptive humor or hold self-perceptions that may diverge from how others perceive them. However, Martin et al. (2003) presented initial validation data that indicated that the HSQ scales have low correlations with social desirability and align well with independent peer ratings of coping behavior. Subsequent cross-cultural validation studies have also confirmed the factor structure and reliability of the instrument (Schermer et al., 2023).

More broadly, Chan (2009) argued that such biases are neither ubiquitous nor uniform across constructs, and that their actual impact is often substantially smaller than assumed. Particularly important for the constructs assessed in the current study, Chan (2009) pointed out that self-report is both justifiable and preferable when assessing constructs that are inherently self-referential and experiential in nature. As humor styles reflect how individuals subjectively orient toward and deploy humor, and PTG refers to a subjective appraisal of personal change, neither construct can be meaningfully assessed by external observers. Nevertheless, future studies could examine whether observer ratings of humor use align with self-reports in predicting PTG.

As the HSQ was completed in English, a specific concern regarding the higher readability demands of the self-enhancing humor subscale, compared to the other HSQ subscales (Silvia & Rodriguez, 2020), should be noted. Future studies could consider utilizing simplified or translated HSQ items in non-English samples, as translated versions of the HSQ have been found to provide adequate assessments of its core constructs (Schermer et al., 2023).

The findings of this study contribute to the understanding of humor as a psychological resource in the context of trauma recovery. Future studies could consider examining the role of humor styles in therapeutic interventions designed to promote PTG or explore whether specific cognitive processes, such as deliberate rumination or benefit-finding, mediate the relationship between self-enhancing humor and PTG.

## 5. Conclusions

This study examined whether adaptive humor styles predicted the five domains of post-traumatic growth in a sample of South African undergraduates. Self-enhancing humor emerged as a significant positive predictor of all five PTG domains, supporting its theoretical description as a cognitive reappraisal resource that could facilitate meaning reconstruction after trauma. Affiliative humor did not contribute unique predictive variance with its apparent associations attributable to shared variance with self-enhancing humor. Pending replication in longitudinal research designs, the findings of this study suggest that self-enhancing humor may play an important role in PTG-related interventions.

## Figures and Tables

**Table 1:** Types of Trauma and Their Frequency (*N* = 194).

Frequency	Percentage	Event
52	26.80	Death of a loved one
52	26.80	Being robbed
37	19.07	Experiencing a serious accident or a serious health problem
15	7.73	Sexual trauma
11	5.67	Serious accident/illness/health problem of a close family member
11	5.67	Physically assaulted/attacked
9	4.64	Witness a violent death/event
6	3.09	Domestic violence
1	0.52	Physical abuse from parent/relative

*Note.* Trauma types were grouped based on participants’ brief open-ended descriptions of their traumatic event.

**Table 2:** Pearson Correlations Among Subscale Composites (*N* = 194).

	*M*	*SD*	ω	α	Affiliative Humor	Self-Enhancing Humor	PTG-I	PTG-II	PTG-III	PTG-IV	PTG-V
Affiliative humor	5.21	0.87	0.82	0.81	—	0.525 ***	0.189 **	0.125	0.108	0.072	0.071
Self-enhancing humor	4.65	1.02	0.83	0.83		—	0.269 ***	0.246 **	0.284 ***	0.266 ***	0.214 **
PTG-I	3.34	0.86	0.82	0.82			—	0.568 ***	0.384 ***	0.281 ***	0.359 ***
PTG-II	3.46	0.82	0.74	0.73				—	0.605 ***	0.473 ***	0.576 ***
PTG-III	3.96	0.79	0.74	0.72					—	0.425 ***	0.526 ***
PTG-IV	3.81	1.08	0.68	0.68						—	0.353 ***
PTG-V	3.93	0.81	0.65	0.63							—

*Note.* ** *p* < 0.01. *** *p* < 0.001 (two-tailed); α = Cronbach’s alpha; ω = McDonald’s omega; PTG-I = Relating to Others; PTG-II = New Possibilities; PTG-III = Personal Strength; PTG-IV = Spiritual Change; PTG-V = Appreciation of Life.

**Table 3:** CFA Standardized Factor Loadings (*N* = 194).

Factor	Indicator	Verbatim Item Text	β	*SE*	*z*
PTG-I: Relating to Others	PTG6	I more clearly see that I can count on people in times of trouble.	0.691	0.051	13.64
	PTG8	I have a greater sense of closeness with others.	0.727	0.044	16.35
	PTG9	I am more willing to express my emotions.	0.592	0.055	10.81
	PTG15	I have more compassion for others.	0.455	0.075	6.09
	PTG16	I put more effort into my relationships.	0.475	0.073	6.51
	PTG20	I learned a great deal about how wonderful people are.	0.714	0.050	14.36
	PTG21	I better accept needing others.	0.727	0.046	15.96
PTG-II: New Possibilities	PTG3	I developed new interests.	0.485	0.072	6.77
	PTG7	I established a new path for my life.	0.694	0.069	10.10
	PTG11	I am able to do better things with my life.	0.707	0.054	13.17
	PTG14	New opportunities are available which wouldn’t have been otherwise.	0.554	0.060	9.19
	PTG17	I am more likely to try to change things which need changing.	0.520	0.066	7.86
PTG-III: Personal Strength	PTG4	I have a greater feeling of self-reliance.	0.498	0.086	5.81
	PTG10	I know better that I can handle difficulties.	0.642	0.059	10.81
	PTG12	I am better able to accept the way things work out.	0.657	0.060	11.01
	PTG19	I discovered that I’m stronger than I thought I was.	0.760	0.043	17.55
PTG-IV: Spiritual Change	PTG5	I have a better understanding of spiritual matters.	0.658	0.074	8.84
	PTG18	I have a stronger religious faith.	0.785	0.079	9.88
PTG-V: Appreciation of Life	PTG1	I changed my priorities about what is important in life.	0.541	0.109	4.94
	PTG2	I have a greater appreciation for the value of my own life.	0.583	0.081	7.21
	PTG13	I can better appreciate each day.	0.669	0.081	8.28
Affiliative Humor	HSQ1	I usually don’t laugh or joke around much with other people.	0.589	0.061	9.61
	HSQ5	I don’t have to work very hard at making other people laugh—I seem to be a naturally humorous person.	0.603	0.063	9.58
	HSQ9	I rarely make other people laugh by telling funny stories about myself.	0.280	0.101	2.78
	HSQ13	I laugh and joke a lot with my closest friends.	0.505	0.075	6.72
	HSQ17	I usually don’t like to tell jokes or amuse people.	0.694	0.060	11.56
	HSQ21	I enjoy making people laugh.	0.837	0.048	17.32
	HSQ25	I don’t often joke around with my friends.	0.649	0.059	10.95
	HSQ29	I usually can’t think of witty things to say when I’m with other people.	0.640	0.059	10.85
Self-enhancing Humor	HSQ2	If I am feeling depressed, I can usually cheer myself up with humor.	0.601	0.073	8.20
	HSQ6	Even when I’m by myself, I’m often amused by the absurdities of life.	0.639	0.063	10.20
	HSQ10	If I am feeling upset or unhappy I usually try to think of something funny about the situation to make myself feel better.	0.748	0.059	12.75
	HSQ14	My humorous outlook on life keeps me from getting overly upset or depressed about things.	0.615	0.063	9.78
	HSQ18	If I’m by myself and I’m feeling unhappy, I make an effort to think of something funny to cheer myself up.	0.738	0.049	14.94
	HSQ22	If I am feeling sad or upset, I usually lose my sense of humor.	0.456	0.068	6.74
	HSQ26	It is my experience that thinking about some amusing aspect of a situation is often a very effective way of coping with problems.	0.586	0.059	9.93
	HSQ30	I don’t need to be with other people to feel amused—I can usually find things to laugh about even when I’m by myself.	0.520	0.071	7.29

*Note.* All loadings *p* < 0.001 except HSQ9 (*p* < 0.005). Standardized loadings estimated with MLR using the original metric. Items (HSQ1, HSQ9, HSQ17, HSQ25, HSQ29 [Affiliative humor]; HSQ22 [Self-enhancing Humor]) were reversed prior to CFA estimation; item text reproduced verbatim from the Posttraumatic Growth Inventory (PTGI; Tedeschi & Calhoun, 1996, p. 460) and the Humor Styles Questionnaire (HSQ; Martin et al., 2003, pp. 58–59).

**Table 4:** Model Fit Indices (*N* = 194).

Model	χ^2^ (*df*)	*p*	CFI	TLI	RMSEA	90% CI	SRMR
Measurement Model (CFA)	893.40 (608)	<0.001	0.870	0.858	0.051	0.043, 0.057	0.074
Structural Model	893.40 (608)	<0.001	0.870	0.858	0.050	0.043, 0.057	0.074

*Note.* χ^2^ = Satorra–Bentler-scaled chi-square statistic; RMSEA = Root Mean Square Error of Approximation; SRMR = Standardized Root Mean Square Residual. Robust fit indices (CFI, TLI, RMSEA) based on MLR estimation.

**Table 5:** Structural Path Coefficients: Affiliative humor and Self-enhancing humor Predicting PTG Dimensions (*N* = 194).

Outcome	Predictor	*B*	*SE*	*z*	*p*	95% CI	β	*R* ^2^
PTG-I: Relating to Others	Affiliative humor	0.086	0.103	0.83	0.405	−0.116, 0.288	0.009	0.109
	Self-enhancing humor	0.304	0.115	2.65	0.008	0.079, 0.529	0.324	
PTG-II: New Possibilities	Affiliative humor	−0.095	0.084	−1.13	0.259	−0.260, 0.070	−0.142	0.154
	Self-enhancing humor	0.279	0.088	3.18	0.001	0.107, 0.451	0.460	
PTG-III: Personal Strength	Affiliative humor	−0.076	0.081	−0.93	0.352	−0.235, 0.084	−0.120	0.155
	Self-enhancing humor	0.258	0.089	2.90	0.004	0.084, 0.433	0.453	
PTG-IV: Spiritual Change	Affiliative humor	−0.118	0.114	−1.03	0.303	−0.342, 0.106	−0.137	0.169
	Self-enhancing humor	0.373	0.102	3.67	<0.001	0.174, 0.572	0.477	
PTG-V: Appreciation of Life	Affiliative humor	−0.080	0.088	−0.92	0.359	−0.252, 0.091	−0.125	0.137
	Self-enhancing humor	0.251	0.079	3.16	0.002	0.095, 0.407	0.430	

*Note. B* = unstandardized coefficient; β = standardized coefficient; *SE* = robust standard error. MLR estimator used throughout. The latent covariance between affiliative and self-enhancing humor was freely estimated (*r* = 0.593, *p* < 0.001).

**Table 6:** Structural Path Coefficients: Affiliative Humor and Self-enhancing Humor Predicting Total PTGI (*N* = 194).

Predictor	*B*	*SE*	*z*	*p*	95% CI	β
Affiliative humor	−0.042	0.066	−0.63	0.531	−0.172, 0.088	−0.064
Self-enhancing humor	0.268	0.071	3.79	<0.001	0.130, 0.407	0.455

*Note.* Outcome = total PTGI latent factor (single error-corrected indicator; residual fixed to 0.047). *B* = unstandardized coefficient; β = standardized coefficient; SE = robust standard error; estimator: MLR. Latent correlation between affiliative humor and self-enhancing humor was freely estimated (r = 0.593). *R*^2^ = 0.177. Model fit: χ^2^(117) = 159.41, *p* = 0.006; CFI = 0.952; TLI = 0.944; RMSEA = 0.045, 90% CI [0.025, 0.061]; SRMR = 0.060.

## Data Availability

The data presented in this study are available on request from the corresponding author due to privacy restrictions.

## Notes

1 Aggressive and self-defeating humor were included in an exploratory supplementary analysis where all four humor styles were entered simultaneously as predictors of the five PTG domains, as well as total PTG. The results are reported in Supplementary Materials. The primary analyses reflect the study’s hypotheses, which were based on the theoretical links between the adaptive humor styles and the reappraisal and meaning-making processes central to PTG.

2 These population groups are described in accordance with the South African census categories.

## Supplementary Materials

The following supporting information can be downloaded at: https://www.mdpi.com/article/10.3390/bs16071131/s1,Table S1: Descriptive Statistics and Reliability Estimates for Maladaptive Humor Subscales (*N* = 194). Table S2: Zero-Order Pearson Correlations Among All Four Humor Styles and PTG Outcomes (*N* = 194). Table S3: Exploratory Structural Path Coefficients: All Four HSQ Styles Predicting PTG Dimensions Simultaneously (*N* = 194).
