# Supplementary material for: Adaptive Humor Styles as Predictors of Post-Traumatic Growth Factors
Source: Behav Sci (Basel). 2026 Jul 6;16(7):1131. doi: 10.3390/bs16071131 (PMC13405941; doi:10.3390/bs16071131)
Supplement: Supplementary file 1 [file behavsci-16-01131-s001.zip › behavsci-4325771-supplementary.pdf]

Supplementary Materials

This supplement presents exploratory analyses that examined whether the inclusion of the aggressive and self-defeating humor styles as additional predictors would alter the primary findings. The original study did not include these humor styles as the focus was on the adaptive humor styles, which had theoretical links with PTG. The supplementary analyses are included here to provide a broader view of the role of the humor styles in PTG.

Table S1. Descriptive Statistics and Reliability Estimates for Maladaptive Humor Subscales (*N* = 194).

Scale	*M*	*SD*	α	ω
Aggressive humor	3.51	0.65	0.429	0.442
Self-defeating	3.52	0.86	0.691	0.703

Note. α = Cronbach's alpha; ω = McDonald's omega.

Table S2. Zero-Order Pearson Correlations Among All Four Humor Styles and PTG Outcomes (*N* = 194).

	1	2	3	4	5	6	7	8	9
1. Affiliative Humor	—								
2. Self-enhancing Humor	0.525***	—							
3. Aggressive Humor	0.178*	−0.008	—						
4. Self-defeating Humor	0.097	0.150*	0.273***	—					
5. PTG-I: Relating to Others	0.189**	0.269***	−0.079	−0.053	—				
6. PTG-II: New Possibilities	0.125	0.246**	−0.099	0.001	0.568***	—			
7. PTG-III: Personal Strength	0.108	0.284***	−0.239***	−0.073	0.384***	0.605***	—		
8. PTG-IV: Spiritual Change	0.072	0.266***	−0.162*	0.043	.281***	0.473***	0.425***	—	
9. PTG-V: Appreciation of Life	0.071	0.214**	−0.126	−0.060	0.359***	0.576***	0.526***	0.353***	—
10. Total PTGI	0.170*	0.338***	−0.168*	−0.044	0.807***	0.866***	0.743***	0.584***	0.686***

Note. * $p < .05$. ** $p < .01$. *** $p < .001$ (two-tailed).

Table S3. Exploratory Structural Path Coefficients: All Four HSQ Styles Predicting PTG Dimensions Simultaneously (*N* = 194).

Outcome	Predictor	*B*	*SE*	*z*	*p*	95% CI	β	R^2
PTG-I: Relating to Others	Affiliative humor	0.047	0.120	0.39	0.694	−0.188, 0.282	0.046	0.122
	Self-enhancing humor	0.340	0.153	2.22	0.027	0.039, 0.641	0.361	
	Aggressive humor	−0.323	0.731	−0.44	0.658	−1.756, 1.110	−0.116	
	Self-defeating humor	0.012	0.213	0.06	0.954	−0.405, 0.430	0.010	
PTG-II: New Possibilities	Affiliative humor	−0.099	0.091	−1.09	0.275	−0.277, 0.079	−0.147	0.165
	Self-enhancing humor	0.278	0.110	2.52	0.012	0.062, 0.495	0.449	
	Aggressive humor	0.136	0.495	0.28	0.784	−0.835, 1.106	0.074	
	Self-defeating humor	−0.121	0.156	−0.78	0.438	−0.425, 0.184	−0.144	
PTG-III: Personal Strength	Affiliative humor	−0.049	0.080	−0.62	0.538	−0.207, 0.108	−0.083	0.191
	Self-enhancing humor	0.249	0.108	2.30	0.021	0.037, 0.462	0.458	
	Aggressive humor	0.065	0.590	0.11	0.912	−1.091, 1.222	0.041	
	Self-defeating humor	−0.142	0.159	−0.89	0.373	−0.454, 0.170	−0.192	
PTG-IV: Spiritual Change	Affiliative humor	−0.118	0.138	−0.85	0.394	−0.388, 0.153	−0.134	0.172
	Self-enhancing humor	0.389	0.145	2.69	0.007	0.106, 0.673	0.483	
	Aggressive humor	−0.032	1.192	−0.03	0.979	−2.367, 2.304	−0.013	
	Self-defeating humor	0.019	0.331	0.06	0.955	−0.629, 0.667	0.017	
PTG-V: Appreciation of Life	Affiliative humor	−0.076	0.094	−0.80	0.422	−0.261, 0.109	−0.116	0.182
	Self-enhancing humor	0.237	0.104	2.28	0.023	0.033, 0.441	0.398	
	Aggressive humor	0.279	0.562	0.50	0.620	−0.824, 1.381	0.158	
	Self-defeating humor	−0.227	0.174	−1.31	0.191	−0.567, 0.113	−0.281	
Total PTGI	Affiliative humor	−0.020	0.069	−0.29	0.774	−0.156, 0.116	−0.031	0.190
	Self-enhancing humor	0.277	0.090	3.08	0.002	0.101, 0.453	0.469	
	Aggressive humor	−0.056	0.481	−0.12	0.907	−0.999, 0.887	−0.033	
	Self-defeating humor	−0.059	0.138	−0.43	0.671	−0.328, 0.211	−0.073	

Note. The total PTGI model used a single error-corrected indicator with residual fixed to 0.047 (model fit: CFI = .734, TLI = .711, RMSEA = .066 [.058, .073], SRMR = .100). *B* = unstandardized coef-

ficient; β = standardized coefficient; *SE* = robust standard error. MLR estimator used throughout; results are exploratory and were not part of the primary analysis. All four humor styles were entered simultaneously as latent predictors; results are exploratory and were not part of the primary analysis.

Aggressive and Self-Defeating Humor Styles as Predictors of the Five PTG Dimensions and Total Score

These analyses are exploratory and were not part of the primary analysis. The PTG subscale model followed the same 5-factor PTG measurement structure as the primary analysis. Affiliative and self-enhancing humor acted as latent factors, as in the primary analysis, with aggressive and self-defeating humor added as additional latent predictors (nine factors total, 69 indicators). Model fit was lower compared to the primary model (CFI = .728, TLI = .709, RMSEA = .058 [.053, .062], SRMR = .090). This outcome was likely due to the poor internal consistency of aggressive humor (α = .429). The path coefficients from aggressive humor should therefore be interpreted with caution.
